# Elimination of human African trypanosomiasis: The long last mile

**DOI:** 10.1371/journal.pntd.0012091

**Published:** 2024-05-01

**Authors:** Michael P. Barrett, Gerardo Priotto, Jose R. Franco, Veerle Lejon, Andreas K. Lindner

**Affiliations:** 1 School of Infection & Immunity, College of Medical, Veterinary and Life Sciences, University of Glasgow, Glasgow, United Kingdom; 2 Department of Control of Neglected Tropical Diseases, World Health Organization, Geneva, Switzerland; 3 UMR177 Intertryp, Institut de Recherche pour le Développement, CIRAD, University of Montpellier, Montpellier, France; 4 Charité - Universitätsmedizin Berlin, Charité Center for Global Health, Institute of International Health, Berlin, Germany; Hebrew University-Hadassah Medical School, ISRAEL

They said the last mile would be the hardest. The challenge to eliminate human African trypanosomiasis caused by *Trypanosoma brucei gambiense* (gHAT) could have entered that most difficult stretch. The fifth stakeholders’ meeting on *gambiense* and *rhodesiense* HAT, convened by the World Health Organisation (WHO) in Geneva (7–9 June 2023) (https://www.who.int/news-room/events/detail/2023/06/07/default-calendar/fifth-who-stakeholders-meeting-on-human-african-trypanosomiasis-elimination), was able to report that the incidence of HAT has remained beneath 1,000 new cases reported annually for the fifth year in a row. A historic achievement given that the numbers were estimated to exceed 300,000 people infected at the turn of this century; the 35,000 or so officially reported in 1997 believed to represent only around 10% of the true numbers based on patchy surveillance at that time [1]. A total of 7 of the 36 countries in sub-Saharan Africa that were once endemic for HAT have now been validated by WHO as having eliminated the disease as a public health problem (defined as there being fewer than 1 person per 10,000 population affected in all health districts of the country over the preceding 5 years). Since the fourth stakeholders’ meeting in 2021, however, the numbers have, actually, risen slightly. Epidemiological modelling [2], along with our knowledge from other infectious diseases entering their end game (e.g., Guinea worm and polio [3]), also point to an inevitable long tail in clearing up the last cases. Nevertheless, HAT has ceased to be a public health problem, thanks to the extraordinary efforts of National Sleeping Sickness Control Programmes, supported by various stakeholders, coordinated by the WHO. And yet, a number of questions inevitably arise in this phase of diminishing returns, where the rate of decline in incidence, as a function of interventional efforts, wanes.

Did the COVID-19 pandemic play a role in hindering progress, for example? Although difficult to quantify, the pandemic had non-negligible impact on the elimination of HAT; intervention programs suffered and case detection was disrupted too.

Some ask whether the HAT elimination campaign is a victim of its own success. Cash-strapped health ministries struggle to justify funds to sustain a program overseeing a low prevalence or even, in some cases, absent disease, while other infectious diseases continue unabated. Donors, too, require enlightened persuasion to stay the course, and the global economic woes stemming from COVID-19 and other geopolitical upheavals don’t help. However, robust surveillance is essential for 5 years following the reporting of the last case and countries must remain capable of detecting cases beyond then, to meet WHO criteria allowing a country to be declared HAT-free [4]. The apprehensions of populations of HAT-endemic nations naturally turn to existing diseases, so health-seeking behaviours change. For healthcare workers, HAT-specialists are ageing, many are now retired and new staff have few opportunities for training in HAT to replace them. For those remaining, sitting around as a specialist for a disease without cases, is not an option; they are moved into other areas. Ultimately, their skills in detection and management of HAT disappear. In this regard—the elimination campaign can indeed be seen to have been a victim of its own success—which is why concerted efforts are required to stay the course.

In previous times of diminishing incidence of HAT, control programs dwindled, and the disease reappeared. This time, though, things seem different. The lessons of the past are known, and action to avert a foreseeable resurgence can be implemented in one last push.

To this backdrop, the fifth stakeholders’ meeting was abuzz with optimism in spite of the stuttering in the decline of HAT incidence. The anticipated availability of new tools, adapted to the new situation, which will enable efforts to be intensified at this critical time, adds to growing optimism.

Success over the past decade or more has depended on 3 pillars: case-detection and treatment with effective medicines, both complemented by control of the tsetse fly vector.

## Medicines for gambiense HAT

Medicines exist to treat HAT. A massive part of the success in combatting the disease has stemmed from ensuring availability of existing drugs to all patients, being donated, free of charge, by manufacturers to the WHO which guarantees distribution to national programs. Sanofi, for example, initially as Aventis, has provided eflornithine for 23 years. Sanofi also donates melarsoprol, the highly toxic arsenic-based drug, which kills up to 5% of patients it is supposed to cure, although it is no longer needed for gambiense HAT, and as discussed later, for rhodesiense HAT it may be replaced by safer compounds in the not too distant future. Pentamidine is another of the generous offerings from Sanofi, who have also picked up the provision of fexinidazole discussed below. German pharmaceutical company Bayer provides suramin for free, and, in a heartening collaboration between potential rivals, they also provide nifurtimox, which is given in combination with Sanofi’s eflornithine for nifurtimox-eflornithine combination therapy (NECT).

The introduction of fexinidazole, an all-oral treatment suitable for both stage 1 and stage 2 treatment (other than where parasites have entered deep within brain parenchyma for which NECT is still needed) [5], has made a significant difference, transforming the logistics of administration by removing the need for a syringe and large solution volumes. Fexinidazole tablets are simpler to deliver to areas requiring the drug, compared to infusion-injected eflornithine, for example, where enormous volumes are needed for each patient. Recent stability data have now shown fexinidazole’s shelf-life can be extended to 5 years, so consignments of pills can be shipped to endemic foci and stockpiled locally for much longer.

Fexinidazole was developed in the early 2000s, after the Drugs for Neglected Diseases initiative (DNDi) picked it up as a compound having shown efficacy in studies in the 1980s but not taken further then. It was eventually registered in 2018, since when all HAT-endemic countries have followed the DRC in bringing it into their pharmacopoeias for use against gHAT. By 2022, half of all HAT patients were being treated with this drug. To date, pharmacovigilance data collected by WHO, confirm fexinidazole to be as safe and as efficacious as trials had suggested. The possibility that fexinidazole will be introduced for treatment of rhodesiense HAT (rHAT) too will offer a further breakthrough if the current necessity of melarsoprol treatment can be superceded. Nevertheless, fexinidazole is still complex to administer (needing coadministration with food, for 10 days and provoking relatively frequent gastrointestinal side effects).

In 2022, acoziborole, an orally available compound that cures both stage 1 and stage 2 gHAT disease with a single dose, demonstrated efficacy in a pivotal phase 2/3 trial [6]. Acoziborole’s safety profile is better than that of fexinidazole. The medicine, also developed by DNDi, following its discovery through Scynexis and Anacor Pharmaceuticals, will also be marketed, when available, by Sanofi. If further trials continue to support acoziborole’s safety and efficacy, plans for a “screen and treat” strategy can be considered. In principle, acoziborole could be given with ease to anyone with a suspicion of HAT (e.g., those testing positive in a serological test even without confirmed diagnosis), provided there is no risk of significant adverse events. Additional work, optimising paediatric use for the new drugs is also underway to offer complete coverage of treatments for all people at risk from HAT.

## Case detection

Considering the limited specificity of serological tests, the side effects of currently available medicines and the current epidemiological status of the disease, parasitological confirmation is recommended to diagnose the disease. The complexity of parasitological diagnosis often means that patients must travel tens of kilometres to a specialist centre. Improved diagnostics are highly desirable.

Serological tests, notably the card agglutination test for trypanosomiasis (CATT) assay, where blood from simple finger pricks is assessed for its ability to agglutinate freeze-dried trypanosomes of particular antigen types on a card have been critical in case-detection for years [7]. Translating the main antigens used in the CATT assay to simplified lateral flow tests, or rapid tests as they are also known, has offered the potential to further facilitate screening, albeit not diagnosis of the disease [8]. Specificity limitations mean that it is currently recommended to find parasites in blood or CSF to confirm positive serological tests. In an environment of decreasing incidence of HAT, where the positive predictive value of any test diminishes, the rapid tests, in spite of their limitations, may be suitable to provide a reasonable suspicion of infection. If improved therapies such as acoziborole continue to show good safety, they may enable “screen and treat” scenarios to become a possibility as individuals falsely testing positive can be given the drug without a fear of side effects.

The limitations of the serological tests, and the possible risk that strains of parasite are in circulation that do not express the antigens that form the basis of these tests, means that better diagnostics remain desirable. A highly specific trypanolysis assay that detects anti-trypanosomal antibodies in blood of patients requires samples to be sent to a highly specialised reference centre, so an inhibition ELISA derivative of that approach is under development [9]. A priori, trypanosome nucleic acids could represent a surrogate for the parasites but be easier to detect. The suitability of parasite DNA has been questioned given its persistence, even after successful treatment [10]. RNA might be more suitable, although its relative instability has, so far, meant that robust RNA-based tests have not yet been forthcoming. Inclusion of nucleic acid preservation reagents in blood collection kits, however, is improving the situation and RNA tests are now being introduced for extensive testing of serological suspects. PCR was the prototypical means of amplifying nucleic acids, followed by the ambient temperature derivative loop-mediated isothermal amplification (LAMP). Other molecular breakthroughs, such as the CRISPR Cas13-based SHERLOCK [11] approach, also bypass the need for a thermal cycling device, although the problems of sensitivity around molecular approaches, in particular to confirm *Trypanosoma brucei gambiense* infection, for now, remain, together with the need to refer samples to specialised labs. The requirement for highly specific and sensitive tests will be essential to verify the true absence of cases in areas where the absence of transmission of the disease is believed to have been achieved. Equally, historical foci of the disease should be kept under surveillance with sensitive tests to assure no resurgence.

## Tsetse control

The third pillar of the elimination campaign, complementing the other elimination tools, is vector control (VC). As case numbers become very low, sustained VC will play a role in some settings [12].

Trypanosomes are transmitted by multiple different species of tsetse fly (genus *Glossina*), and both male and female tsetse can carry the parasites. The different vector species have different physiological and behavioural characteristics, which preclude a simple one-size-fits-all approach to tsetse control.

However, the contribution of insecticide spraying, nowadays preferably as targeted ground spraying, although indiscriminate aerial spraying does retain utility in some places, can selectively remove tsetse from a given locality. Concerns on environmental impacts of insecticide use need to be considered at all times. More sophisticated routes to trap flies or to target them through insecticide-impregnated “Tiny targets,” or other types of target, have also had great success where used rationally [13]. Applying insecticides to large mammals, particularly cows as live bait that attract tsetse, plays an important role too.

The release of irradiated, and thus sterile, male *Glossina*, was successful in Zanzibar and has been trialled elsewhere [14]. The costs and sociological hurdles to the release of irradiated flies, however, plus biological limitations on species-restricted mating capability, continue to constrain the sterile male approach. The International Atomic Energy Agency (IAEA), however, remains a key stakeholder in the elimination campaign and continues to seek ways to deliver tsetse control to the continent.

An understanding of the relative role of infected tsetse numbers in transmission can assist in knowing where to target vector control measures. Covering the entire tsetse belt with traps or targets at a density where they can impact on numbers is not feasible. However, by collecting epidemiological data and using it to implement tsetse control campaigns selectively in highly affected areas or in places with frequent human–tsetse contact, offers a realistic proposition.

The intersection of programs against both human and animal African trypanosomiasis through tsetse control offers an opportunity to combat both problems through a “One Health” approach, with sharing of tools and know-how as well as strategic planning and stakeholder collaboration.

## Turning to Rhodesiense HAT

The campaign to eliminate transmission of human African trypanosomiasis has focused primarily on the disease caused by *Trypanosoma brucei gambiense* which is, for the most part, confined to human hosts without a significant animal reservoir, although reports of possible infections in domestic animals, including pigs, and wild animals cannot be excluded [15]. In the case of the disease caused by *T*. *b*. *rhodesiense*, interruption of transmission has been considered unrealistic given the ability of these parasites to infect a wide range of domestic and wild animals. What is more, as recently revealed in Ethiopia [16], the disease can flare up in regions from which it has been absent for a significant period (31 years in the case of Ethiopia). Malawi and Zambia too have also suffered recent outbreaks. *T*. *b*. *rhodesiense* is, essentially, a human infectious mutant form of nonhuman infectious *T*. *b*. *brucei* [17] and it is also possible that human serum-resistant *T*. *b*. *brucei* mutant parasites can, theoretically, emerge afresh at any time, although this has not been shown in spite of *T*. *b*. *brucei* covering huge areas of Africa. Trials of fexinidazole against this form of the disease in east and southern Africa have recently shown the drug to be efficacious and fexinidazole is expected to replace melarsoprol. Acoziborole may well prove efficacious too but clinical trials are needed.

Notwithstanding, the declining incidence of rHAT, coupled with the availability of safer medicines, along with innovations in tsetse control and intervening in the animal reservoir of rHAT, is strengthening the view that a campaign to eliminate rHAT could now be a realistic proposition. Rapid serological tests, such as those that have contributed substantially to the gHAT campaign have not been developed for rHAT. The progressive disappearance of microscopy in diagnosis, e.g., with serological tests becoming dominant in malaria detection, represents a risk to our ability for detection of the disease where dedicated HAT testing is not employed.

## Integrated control

As with all infectious disease transmission, the concept of R_0_, the basic reproductive number, is central, where it describes the number of new cases spread from each existing case. If R_0_ can be brought below 1, then diseases tend to elimination. It is probable that in most HAT foci, currently, this is already the case. The best way to continue downward pressure is the multidisciplinary approach where case finding and management, brings host numbers down and tsetse control diminishes vector numbers and transmission. Moving HAT management from specialised clinics into regular health facilities is a natural trajectory now that numbers are low and interventions easier than in the past.

In all, the campaign for the elimination as a public health problem of human African trypanosomiasis remains an ongoing success and a model of the ability of multiple stakeholders with targeted coordination to deliver a complex and multifaceted system capable of eliminating a once great scourge of humankind. With the new tools currently in development, there is a reasonable expectation for further advance in the elimination, while also implementing ways to overcome the hurdles that have thwarted this goal in the past.
